# Supplementary material for: Content-rich biological network constructed by mining PubMed abstracts
Source: BMC Bioinformatics. 2004 Oct 8;5:147. doi: 10.1186/1471-2105-5-147 (PMC528731; doi:10.1186/1471-2105-5-147)
Supplement: Additional File 2 — The original results of the above study (non-essential files are deleted to keep the file size under the limit set by BMC bioinformatics). [file 1471-2105-5-147-S2.bz2 › chilibotAdditionalFile2/dip05/716ID11744735E18053/html/CCMF_CCMH.html]

 


 **CCMF** and **CCMH** 
  
Found 8 abstracts in PubMed, retrieved 05.  
 

 What does Google say? 
 PDF only 
| .edu only 

---

**Interactive relationship** (e.g. stimulation, inhibition, etc)

**Inhibitory relationship**- The requirement of both  **CcmF**  and  **CcmH**  for the reduction of the disulphide bond in CcmG indicates that CcmG functions later than  **CcmF**  and  **CcmH**  in cytochrome c assembly, rather than in electron transfer from the membrane associated DipZ also known as DsbD to  **CcmH** .  Ref: 11256948 Biochem J, 2001
**Neutral relationship**- **CcmF**  forms a complex with the heme chaperone CcmE and  **CcmH**  but not with apocytochrome c.  Ref: 11744735 J Biol Chem, 2002
- Co immunoprecipitation experiments revealed that  **CcmF**  interacts directly with the heme donor CcmE and with  **CcmH**  but not with apocytochrome c.  Ref: 11744735 J Biol Chem, 2002
- CcmE is a periplasmic heme chaperone that binds heme covalently and transfers it onto apocytochrome c in the presence of  **CcmF** , CcmG, and  **CcmH** .  Ref: 10339610 Proc Natl Acad Sci U S A, 1999

**Non-interactive relationship** (e.g. studied together, co-existance, homology, etc.)

- Strains lacking  **CcmH**  or both  **CcmF**  and  **CcmH**  are unable to grow under anaerobic conditions where c type cytochromes are required, demonstrating their critical role in the assembly of these electron carriers.  Ref: 12511487 J Bacteriol, 2003
- Its postulated function is to reduce disulphide bonds formed between correctly paired cysteine residues in the cytochrome c apoproteins prior to haem attachment by  **CcmF**  and  **CcmH** .  Ref: 11256948 Biochem J, 2001
- Consistent with this observation, strains lacking both  **CcmF**  and  **CcmH**  are deficient in c type cytochromes when assayed under permissive growth conditions.  Ref: 12511487 J Bacteriol, 2003
- The properties of R. sphaeroides cells containing translational fusions between  **CcmF**  and  **CcmH**  and either Escherichia coli alkaline phosphatase or beta galactosidase suggest that they are each integral cytoplasmic membrane proteins with their presumed catalytic domains facing the periplasm.  Ref: 12511487 J Bacteriol, 2003
- The oxidized form of CcmG accumulated not only in trxA or dipZ mutants defective in the transfer of electrons from the cytoplasm for disulphide isomerization and reduction reactions in the periplasm, but also in  **ccmF**  and  **ccmH**  mutants.  Ref: 11256948 Biochem J, 2001
- In this study, the in vivo function and properties of two cytochrome c maturation proteins,  **CcmF**  and  **CcmH**  from Rhodobacter sphaeroides, were analyzed.  Ref: 12511487 J Bacteriol, 2003
